# Bimetallic FeO_*x*_–MO_*x*_ Loaded TiO_2_ (M = Cu, Co) Nanocomposite Photocatalysts for Complete Mineralization of Herbicides

**DOI:** 10.1021/acs.jpcc.2c06796

**Published:** 2023-01-17

**Authors:** Ayoola Shoneye, Haimiao Jiao, Junwang Tang

**Affiliations:** † Department of Chemical Engineering University College London Torrington Place, London, WC1E 7JE, U.K.

## Abstract

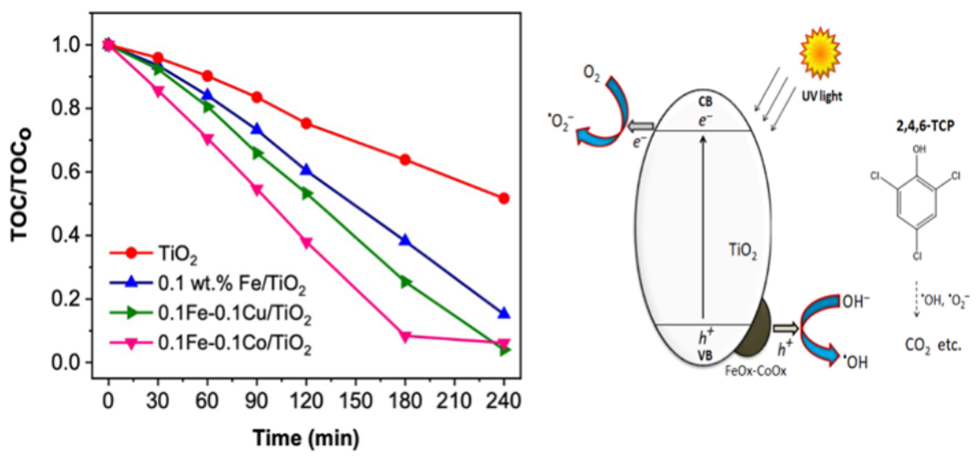

A series of monometallic and bimetallic cocatalyst(s), comprising FeO_*x*_, CuO_*x*_, CoO_*x*_, FeO_*x*_–CuO_*x*_, and FeO_*x*_–CoO_*x*_ loaded TiO_2_ catalysts prepared by the surface impregnation method, were investigated for the photocatalytic mineralization of the widely used four herbicides: 2,4-dichlorophenol (2,4-DCP), 2,4,6-trichlorophenol (2,4,6-TCP), 2,4-dichlorophenoxyacetic acid (2,4-D), and 2,4,5-trichlorophenoxyacetic acid (2,4,5-T). It was found that FeO_*x*_–CoO_*x*_/TiO_2_ showed the highest photocatalytic efficiency toward mineralization of selected herbicides. FeO_*x*_–CoO_*x*_/TiO_2_ achieves 92% TOC removal in 180 min, representing nearly three time activity of the benchmark PC50 TiO_2_. From XPS analysis, FeOOH, CuO, and CoO were determined to be loaded onto the TiO_2_ surface. The outstanding photocatalytic performance of the optimized FeO_*x*_–CoO_*x*_/TiO_2_ sample for herbicides mineralization is due to an increased charge separation and enhanced hydroxyl radicals production monitored by diverse spectroscopies. Based on the proposed charge transfer mechanism, FeO_*x*_–CoO_*x*_ cocatalyst species accelerate the transfer of photogenerated holes on TiO_2_, thus facilitating hydroxyl radicals production.

## 1 Introduction

Water pollution by toxic and persistent pollutants is a growing concern globally due to the increase in world population and the corresponding high demand for clean water for domestic and commercial use. It was reported that over 3.3 billion people (representing over 40% of the world population) lack access to clean water or live in water-stressed areas.^1−3^ Chlorophenols and chlorinated herbicides are major sources of pollution among various organic water contaminants.^4^ These pollutants are released into the environment (surface and underground waters) due to agricultural, nonagricultural, and industrial activities.^4,5^ Most of these environmental persistent chlorinated herbicides are considered toxic or potentially carcinogenic to human and aquatic life and are listed among priority pollutants by US EPA.^6^

Advanced oxidation processes, such as TiO_2_ photocatalysis, is gaining considerable attention as a reliable alternative to conventional water treatment technologies, for the safe destruction of persistent organic water pollutants.^7−9^ TiO_2_ is the most reported material for photocatalytic degradation of organic water pollutants. This is due to its low cost, high stability, and low toxicity to human and aquatic lives.^9−11^ Apart from poor visible light activity, one of the major challenges with TiO_2_ photocatalysis is the low charge separation efficiency of photogenerated electrons and holes, which dominates the overall photocatalytic efficiency for the degradation of targeted organic pollutants.

Surface modification of TiO_2_ with earth-abundant cocatalysts, such as iron, copper, and cobalt oxides, to enhance photocatalytic degradation of chlorinated herbicides, has been widely reported in the literature.^12−14^ Unlike the widely reported codoping of TiO_2_ with Fe and/or Cu as dopants,^15−17^ the use of binary cocatalysts on surface modification of TiO_2_ for photocatalytic water treatment was relatively less reported. A binary codeposition of Ag and Cu nanoparticles on TiO_2_, prepared via photodeposition, was reported for photocatalytic degradation of methylene blue dye and salicylic acid under UV and visible light irradiation.^18^ The Ag–Cu–TiO_2_ sample showed higher degradation efficiency compared to the monometallic (Ag or Cu) deposited TiO_2_. The observed enhancement in photocatalytic activity was reported to be due to extended optical absorption, as a result of surface plasmonic effect. In addition, charge separation is more efficient compared to monometallic (Ag/Cu)–TiO_2_ samples, as revealed by photoluminescence spectra measurements. A bi-co-catalyst alloy involving CuO and CoO (1 wt % Cu and 1 wt % Co) has been reported to improve the hydrogen evolution rate of SnO_2_@TiO_2_ in photocatalytic water splitting under UV light irradiation.^19^ Also, CuO exhibited higher activity than CoO regarding single cocatalyst-decorated samples. The enhancement in H_2_ evolution rate by CuO–CoO/SnO_2_@TiO_2_ was attributed to the synergistic improvement in the transfer of photogenerated electrons by CuO for reduction reaction and holes by CoO for oxidation reaction on the catalyst’s surface. In another study involving single cocatalysts comparison, CuO_*x*_ was reported to display higher activity compared to FeO_*x*_ for the photocatalytic degradation of 4-chlorophenol (4-CP) in aqueous solution with TiO_2_ under UV light irradiation.^20^ Accordingly, constructing bimetallic TiO_2_ based photocatalysts provides a very promising strategy to improve the photocatalytic efficiency in the field of photocatalytic degradation of chlorinated herbicides, benefiting from the desired advantages of bimetallic species such as their synergetic effects and electronic interactions within dual metal species.^21^ Moreover, the development of efficient non-noble bimetallic cocatalysts is also preferable, which could greatly reduce the cost of photocatalysts. In one of our recent reports, FeOOH loaded TiO_2_ exhibited superior performance in photocatalytic herbicides mineralization.^12^ Thus, the combination of Fe and other low cost metal species such as Co and Cu as bimetallic cocatalysts to further enhance the efficiency of photocatalytic mineralization of herbicides is scarcely reported and is worthy to be thoroughly studied.

Herein, novel nanoarchitecture comprising binary metal oxides/hydroxides as cocatalysts on PC50 (commercial benchmark anatase TiO_2_), were synthesized using a reproducible surface impregnation method. The Fe(III), Cu(II), and Co(II) species were characterized in order to clarify their functionality and actual active species. The photocatalytic degradation of 2,4,6-TCP and 2,4-D in water were carried out under UV/vis light irradiation. The effects of single cocatalysts, the dual cocatalyst coupling containing FeOOH as reported earlier,^12^ the nature of the herbicide, and the light wavelength were investigated. Photocatalytic mineralization ability of the optimized catalyst was also evaluated with other widely used herbicides, 2,4-DCP and 2,4,5-T, to demonstrate its wide applications. The charge transfer mechanism was also discussed.

## 2 Experimental Section

### 2.1 Chemicals

PC50 TiO_2_ (purely anatase) was purchased from Millennium Chemicals. 2,4,6-Trichlorophenol (98%) was purchased from Alfa Aesar. 2,4-Dichlorophenoxyacetic acid was purchased from Cayman Chemical Company. 2,4-Dichlorophenol (99%) and coumarin were purchased from Acros Organics. 2,4,5-Trichlorophenoxyacetic acid, Fe(NO_3_)_3_.9H_2_O, Cu(NO_3_)_2_·2.5H_2_O, and Co(NO_3_)_2_·6H_2_O were purchased from Sigma-Aldrich. 2-Propanol (HPLC grade) and acetonitrile (HPLC grade) were purchased from Fisher Scientific. All reagents were used as received without further purification.

### 2.2 Sample Preparation

A modified surface impregnation and drying technique was used to prepare dual cocatalyst-decorated TiO_2_ composites.^12,22^ In a typical experiment, the appropriate percentage weight of the nitrate precursors of Fe(III), Cu(II), and Co(II) was separately added to an aqueous suspension of 1.0 g of commercial PC50 TiO_2_ under mild stirring, with M/TiO_2_ composition (M is the metal with a weight percentage of 0.05, 0.1 and 0.5). The obtained slurry was continuously stirred with a magnetic stirrer bar and dried slowly at 80 °C on a hot plate. The resultant dried powder was hand-milled and calcined in a muffle furnace under air atmosphere at 250 °C for 4 h. It was collected after cooling to room temperature, washed, dried and hand-milled again, and stored for photocatalytic activity tests and characterizations. Subsequent studies on cocatalyst coupling (FeO_*x*_–CuO_*x*_/TiO_2_ and FeO_*x*_–CoO_*x*_/TiO_2_) were evaluated using optimum cocatalyst loading (0.1 wt % for each of the three cocatalysts), followed by a study on the photocatalytic performance of optimized photocatalyst for the mineralization of the four herbicides (2,4-DCP, 2,4,6-TCP, 2,4-D, and 2,4,5-T) under similar operating conditions.

### 2.3 Characterization of Photocatalysts

X-ray photoelectron spectroscopy (XPS) was performed by a Thermo Scientific K-alpha/NEXSA photoelectron spectrometer using monochromatic Al Kα radiation (1486.6 eV); peak positions were calibrated to carbon (284.5 eV) and plotted using the CasaXPS software. UV–vis absorption spectra measurements of powdered samples were performed using a Shimadzu UV-2550 spectrophotometer fitted with an integrating sphere. Inductively coupled plasma atomic emission spectroscopy (ICP-AES) was performed using Varian 720 to investigate the metal leaching. Hydroxyl radicals generated were quantified using aqueous coumarin solution and a Shimadzu RF-6000 Spectrofluorometer.

### 2.4 Photocatalytic Measurements

A 300 W Xe lamp (Newport) was used as the light source with a plain glass window (λ > 320 nm) as a cutoff filter. The glass window shields all UV light with wavelength <320 nm. In a typical measurement, 0.1 g of photocatalyst was dispersed in 200 mL of herbicide solution prepared with deionized water. The suspension was sonicated in an ultrasonic water-bath for 15 min and then magnetically stirred in the dark for 1 h to achieve adsorption/desorption equilibrium of herbicides on the photocatalyst. Thereafter, the suspension was irradiated with UV/visible light under continuous magnetic stirring, while the reaction vessel was immersed in a water-bath to regulate temperature (*T* ≤ 30 °C). Upon light irradiation, a 3 mL sample portion was taken at regular time intervals and filtered through a micropore syringe filter (PTFE, 0.2 μm) before further analysis. Further studies with the best cocatalyst-decorated TiO_2_ sample were carried out using 25 ppm 200 mL solution of four (4) different herbicides: 2,4,6-trichlorophenol (2,4,6-TCP), 2,4-dichlorophenol (2,4-DCP), 2,4-dichlorophenoxyacetic acid (2,4-D), and 2,4,5-trichlorophenoxyacetic acid (2,4,5-T).

### 2.5 Analyses

The change in herbicide concentration was measured using a high performance liquid chromatograph (HPLC-2030C, Shimadzu) consisting of a binary pump, an autosampler, a photodiode array detector and an ACE-5 C18 (5 μm × 150 mm × 4.6 mm) reverse phase column maintained at 40 °C. The HPLC used a 5–95% gradient (acetonitrile/H_2_O with 0.1% formic acid) as the mobile phase.^12^ A UV–vis spectrophotometer was also used to monitor herbicides degradation over the optimized sample. The further herbicides mineralization by photocatalysis was investigated primarily using a Shimadzu total organic carbon (TOC-L) analyzer.

## 3 Results and Discussion

### 3.1 Characterization of FeO_*x*_–MO_*x*_/TiO_2_ Nanocomposites

**Figure 1:**
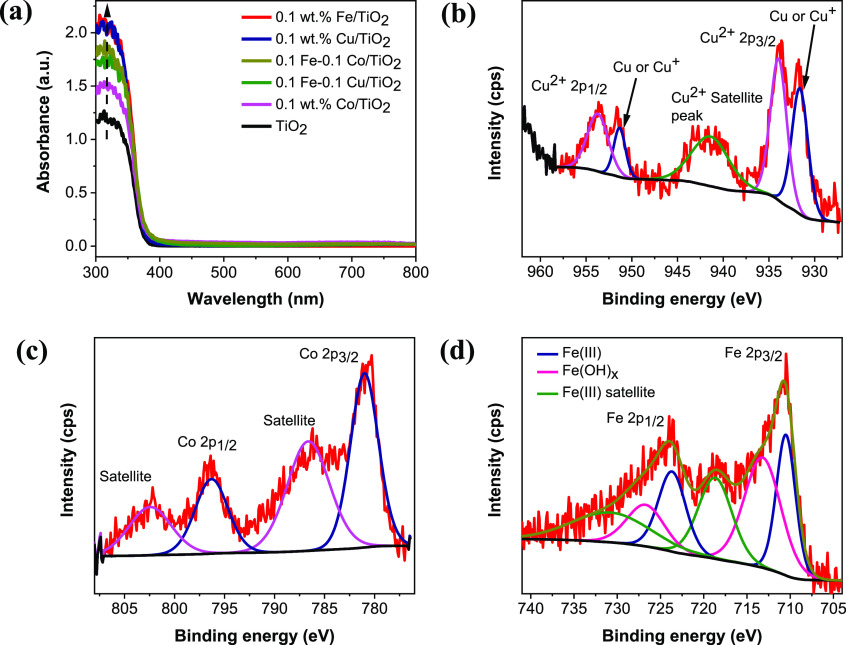
(a) UV/vis absorption spectra of prepared cocatalyst-decorated PC50 TiO_2_ catalysts, (b) Cu 2p XPS spectra of FeO_*x*_–CuO_*x*_/TiO_2_ sample with optimum Cu loading (0.1 wt % Cu and 0.1 wt % Fe), (c) Co 2p XPS spectra of FeO_*x*_–CoO_*x*_/TiO_2_ sample with high Co loading (0.5 wt % Co and 0.5 wt % Fe), and (d) Fe 2p XPS spectra of FeO_*x*_–CoO_*x*_/TiO_2_ sample with high Fe loading (0.5 wt % Fe and 0.5 wt % Co).

The absorption spectra of PC50 TiO_2_ and the cocatalyst-decorated TiO_2_ nanocomposites are shown in Figure 1a. Since the PC50 TiO_2_ is white in color, it does not show any absorption in the visible region with a band gap of 3.2 eV. On the other hand, when dramatically increasing the cocatalyst loading amount, the photocatalysts represent somewhat color. For example, iron oxide-modified PC50 TiO_2_ samples have light-yellow color that indicates the presence of Fe(III) oxide species,^22,23^ which becomes reddish-brown with increase in FeO_*x*_ loading. The cobalt oxide modified PC50 TiO_2_ samples have olive-green or gray color that indicates the presence of CoO,^13^ which also becomes darker with increase in CoO loading. The copper oxide modified PC50 TiO_2_ samples have light blue-green color, indicating the presence of CuO,^24^ which also becomes gray and finally black with increase in CuO loading.

The XPS was used to identify the Fe, Cu and Co oxide species present in the FeO_*x*_–CuO_*x*_/TiO_2_ and FeO_*x*_–CoO_*x*_/TiO_2_ samples. Fe 2p, Cu 2p, and Co 2p peaks are very small observed in the full XPS survey spectrum of both samples (Figure S1), which is likely due to the low amount of cocatalysts loading and high dispersion on TiO_2_ surface.^13,20^ From the fitting Cu 2p XPS spectrum of FeO_*x*_–CuO_*x*_/TiO_2_ in Figure 1b, peaks corresponding to Cu^2+^ are confirmed at 934 eV (Cu 2p_3/2_) and 954 eV (Cu 2p_1/2_).^25,26^ A satellite peak characteristic of the presence of Cu^2+^ is clearly observed between 939–945 eV.^25^ Apart from the Cu^2+^ satellite, the peak at binding energy of 931.6 eV is assigned to Cu/Cu^+^.^14,26^ However, it is difficult to differentiate between Cu_2_O and Cu metal from the Cu 2p XPS peaks as their binding energies are too close and it is also difficult to identify them by other techniques due to very low amount loaded.^14,27^ The Co 2p XPS spectrum of FeO_*x*_–CoO_*x*_/TiO_2_ is of weak intensity due to low Co concentration in the sample as shown in Figure S2. Based on the fitting Co 2p XPS spectrum of FeO_*x*_–CoO_*x*_/TiO_2_ (with high Co loading, 0.5 wt %) in Figure 1c, peaks corresponding to Co^2+^ are confirmed at 781 eV (Co 2p_3/2_) and 796 eV (Co 2p_1/2_).^26,28^ The shakeup satellite features of Co^2+^ at 787 and 802 eV are very strong in intensity, which rules out the presence of Co^3+^, whose satellite features are very weak in intensity at similar binding energies.^29,30^ Also, the Fe 2p core-level XPS spectrum of FeO_*x*_–CoO_*x*_/TiO_2_ is of weak intensity due to low Fe concentration in the sample as shown in Figure S3. Based on the fitting Fe 2p XPS spectrum of FeO_*x*_–CoO_*x*_/TiO_2_ (with high Fe loading, 0.5 wt %) in Figure 1d, peaks corresponding to Fe^3+^ are confirmed at 710 eV (Fe 2p_3/2_) and 724 eV (Fe 2p_1/2_).^26,31−33^ The satellite peaks at 719 and 733 eV are associated with the fingerprint of Fe(III) oxidation state.^32,33^ The extra peaks at 713 and 728 eV are related to the influence of hydroxide groups.^32,33^ Absence of a peak at 709 eV rules out the presence of Fe^2+^ in the sample.^31,34^ Based on the Ti 2p core-level XPS spectra of TiO_2_, FeO_*x*_/TiO_2_, FeO_*x*_–CuO_*x*_/TiO_2_, and FeO_*x*_–CoO_*x*_/TiO_2_ in Figure S4, peaks corresponding to Ti^4+^ are confirmed around 458 eV (Ti 2p_3/2_) and 464 eV (Ti 2p_1/2_).^25,31^ A slight peak shift to lower energy is observed with FeO_*x*_/TiO_2_, while a slight shift to higher energy is observed with FeO_*x*_–CuO_*x*_/TiO_2_ and FeO_*x*_–CoO_*x*_/TiO_2_ compared to pristine TiO_2_. The observation suggests that there is a form of strong interaction between TiO_2_ and the dual cocatalysts, which is neither Ti^4+^ reduction nor oxidation.^32^

### 3.2 Photocatalytic Mineralization of Herbicides

**Figure 2:**
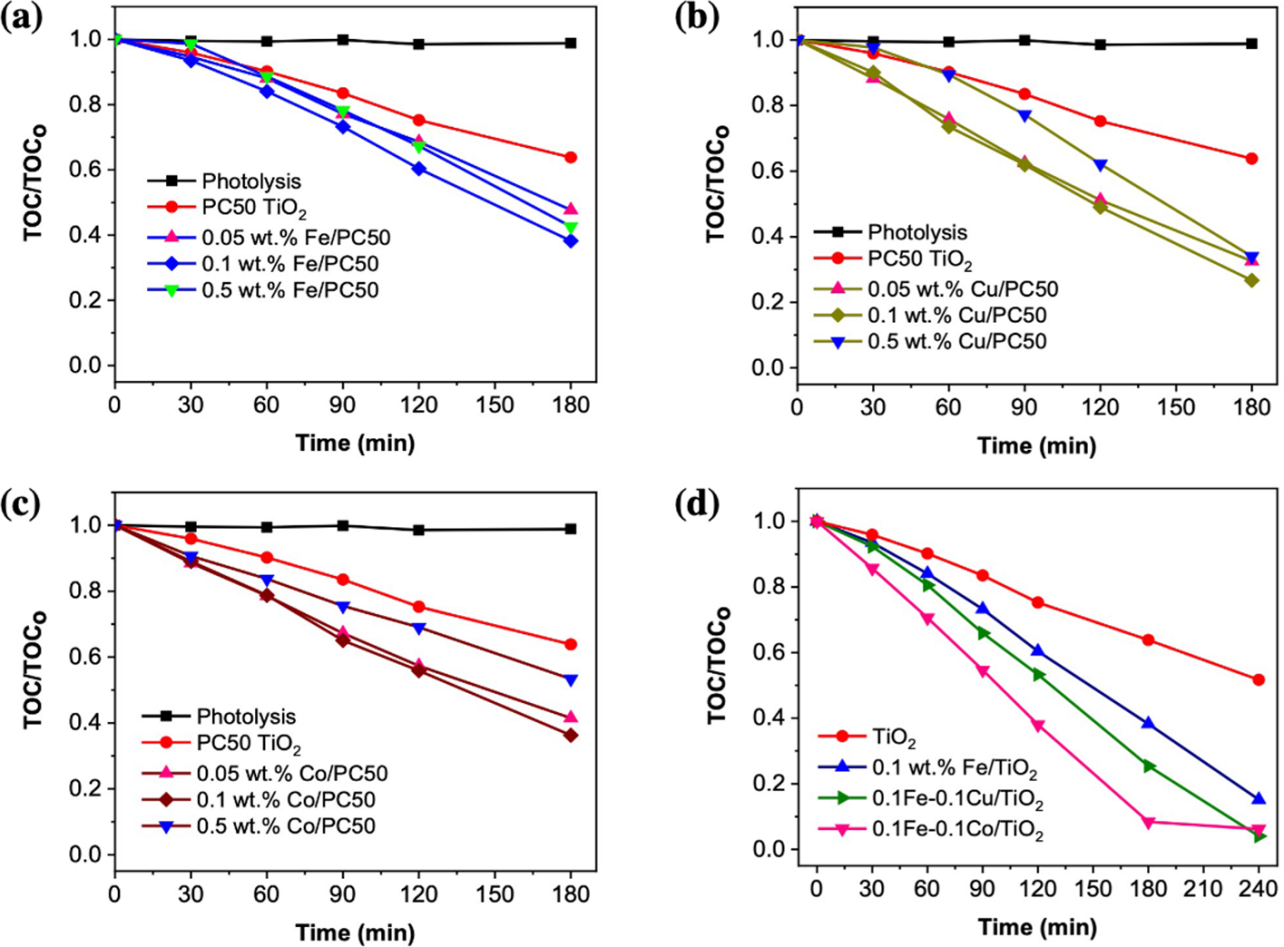
(a) Mineralization profiles of 2,4,6-TCP using FeO_*x*_/TiO_2_ with different Fe loading and control experiments, (b) mineralization profiles of 2,4,6-TCP using CuO_*x*_/TiO_2_ with different Cu loading and control experiments, (c) mineralization profiles of 2,4,6-TCP using CoO_*x*_/TiO_2_ with different Co loading and control experiments, (d) mineralization profiles of 2,4,6-TCP when using FeO_*x*_ as the primary cocatalyst and CuO_*x*_ or CoO_*x*_ as a second cocatalyst. The mineralization profiles were monitored by a TOC analyzer. Conditions: 2,4,6-TCP, 50 ppm, 200 mL, pH = 6, catalyst concentration = 0.5 g/L, and λ > 320 nm.

First, the control experiment was carried out with only 2,4,6-TCP solution in the absence of a photocatalyst. Virtually 0% TOC removal is observed after 3 h of light irradiation as shown in Figure 2a. Photocatalytic activities of the as-prepared mono and dual cocatalyst-decorated TiO_2_ composites were then evaluated by the mineralization of 2,4,6-TCP under full arc light irradiation (λ > 320 nm).

The 2,4,6-TCP mineralization rate recorded on PC50 TiO_2_ is nearly 35% after 3 h. Approximately 52% TOC removal is achieved with the optimized 0.05 wt % Fe/TiO_2_ sample after 3 h. An increase in Fe concentration up to 0.1 wt % leads to a further increase in photocatalytic activity, while lower 2,4,6-TCP mineralization rate is observed with 0.5 wt % Fe-loaded sample. The optimum condition for the preparation of FeO_*x*_/TiO_2_ nanocomposites is found to be 0.1 wt % Fe with ca. 62% TOC removal after 3 h. Similar trends were observed for Co and Cu concentrations as shown in Figures 2b and 2**c**. The optimum condition for the preparation of CuO_*x*_/TiO_2_ and CoO_*x*_/TiO_2_ nanocomposites are found also to be 0.1 wt % Cu and 0.1 wt % Co, with about 73% and 64% TOC removal after 3 h, respectively. An increase in the amount of cocatalyst (0.5 wt %) beyond the optimum negatively affects the photocatalytic activity.

This observation could be due to shielding of intrinsic light absorption by colorful cocatalysts and occupying the oxidation sites on TiO_2_.^22^ For the mono cocatalyst-loaded TiO_2_ samples, the order of photocatalyst activity is CuO_*x*_ > CoO_*x*_ ≈ FeO_*x*_.

Next, the effect of dual cocatalyst loading, e.g., FeO_*x*_–CuO_*x*_ and FeO_*x*_–CoO_*x*_ was investigated based on optimum loading amount for individual cocatalyst (0.1 wt %). A graphical summary showing the influence of CuO_*x*_ on pristine TiO_2_ and FeO_*x*_/TiO_2_ is displayed in Figure S5. The presence of CuO_*x*_ enhances the photocatalytic activity of both samples. About 73% and 75% TOC removal in 3 h are recorded with CuO_*x*_/TiO_2_ and FeO_*x*_–CuO_*x*_/TiO_2_ samples, respectively for 2,4,6-TCP decomposition. A graphical summary showing the influence of CoO_*x*_ on pristine TiO_2_ and FeO_*x*_/TiO_2_ is displayed in Figure S6. CoO_*x*_ also enhances the photocatalytic activity of both samples as observed with CuO_*x*_. However, there is a remarkable enhancement in 2,4,6-TCP mineralization efficiency with the FeO_*x*_–CoO_*x*_/TiO_2_ sample as it records about 92% TOC removal in 3 h. The influence of CuO_*x*_ and CoO_*x*_ on FeO_*x*_/TiO_2_ is clearly compared in Figure 2d. The photocatalytic 2,4,6-TCP mineralization efficiencies in 3 h follow the order: TiO_2_ (36%) < FeO_*x*_/TiO_2_ (62%) < FeO_*x*_–CuO_*x*_/TiO_2_ (75%) < FeO_*x*_–CoO_*x*_/TiO_2_ (92%).

**Figure 3:**
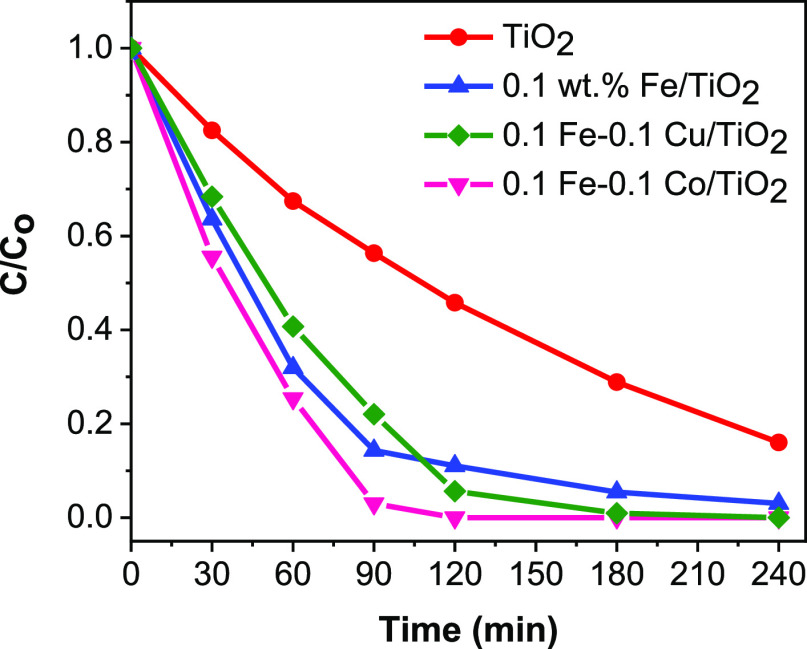
Conversion profiles of 2,4,6-TCP using TiO_2_, FeO_*x*_/TiO_2_, FeO_*x*_–CuO_*x*_/TiO_2_, and FeO_*x*_–CoO_*x*_/TiO_2_ samples monitored by HPLC. Conditions: 2,4,6-TCP, 50 ppm, 200 mL, pH = 6, catalyst concentration = 0.5 g/L, λ > 320 nm.

The 2,4,6-TCP degradation rates over TiO_2_, FeO_*x*_/TiO_2_, FeO_*x*_–CuO_*x*_/TiO_2_, and FeO_*x*_–CoO_*x*_/TiO_2_ from HPLC measurements are compared in Figure 3. The 2,4,6-TCP degradation rates well agree with the 2,4,6-TCP mineralization rates, with the exception of FeO_*x*_–CuO_*x*_/TiO_2_, as it performed less than FeO_*x*_/TiO_2_ in the first 90 min. The photocatalytic 2,4,6-TCP degradation efficiencies in 180 min follow the order: TiO_2_ (71%) < FeO_*x*_/TiO_2_ (95%) < FeO_*x*_–CuO_*x*_/TiO_2_ (99%) < FeO_*x*_–CoO_*x*_/TiO_2_ (100%). Based on above results from HPLC and TOC measurements, FeO_*x*_–CoO_*x*_/TiO_2_ shows comparable efficiency in both photocatalytic 2,4,6-TCP degradation (100%) and mineralization process (92%) in 180 min. Besides, it can see that the degradation rates of 2,4,6-TCP over different photocatalysts decrease continuously in the first 120 min, possibly due to the continuous decreasing pollutant concentration.^35^ In contrast, the mineralization rates by TOC analysis over various samples gradually increases as the reaction progress at the same reaction time. Nevertheless, the 2,4,6-TCP degradation process (monitored by HPLC) is much faster over FeO_*x*_, FeO_*x*_–CuO_*x*_, and FeO_*x*_–CoO_*x*_–decorated TiO_2_ samples than its mineralization. It is because that it starts with fast partial oxidation of the benzene ring to form other organic products as intermediates, while the mineralization process is slow as the final step, which involves the complete breaking of the benzene ring to liberate carbon dioxide (CO_2_).^36−38^ An increase in the photocatalytic activities of the bi-co-catalysts-decorated TiO_2_ samples compared to FeO_*x*_-decorated TiO_2_, is likely due to some synergistic effect from the cocatalyst alloys, which favors efficient separation of photogenerated electrons and holes during photocatalytic degradation of 2,4,6-TCP.

Fe(III) oxide species (mainly FeOOH as proved in our previous study^12^) have been reported to favor the improved generation of hydroxyl radicals as a hole acceptor in the literature.^12,39,40^ It has been reported that Cu(II) oxide species facilitate the transfer of photogenerated electrons on surface of TiO_2_ by enhancing oxygen reduction to generate superoxide radicals.^20,25^ CoO_*x*_ has been reported to be a very good oxidation cocatalyst in photo-oxidation of organic compounds^41,42^ and oxygen evolution in photocatalytic water splitting by improving the lifetime of photogenerated holes.^43−45^

**Figure 4:**
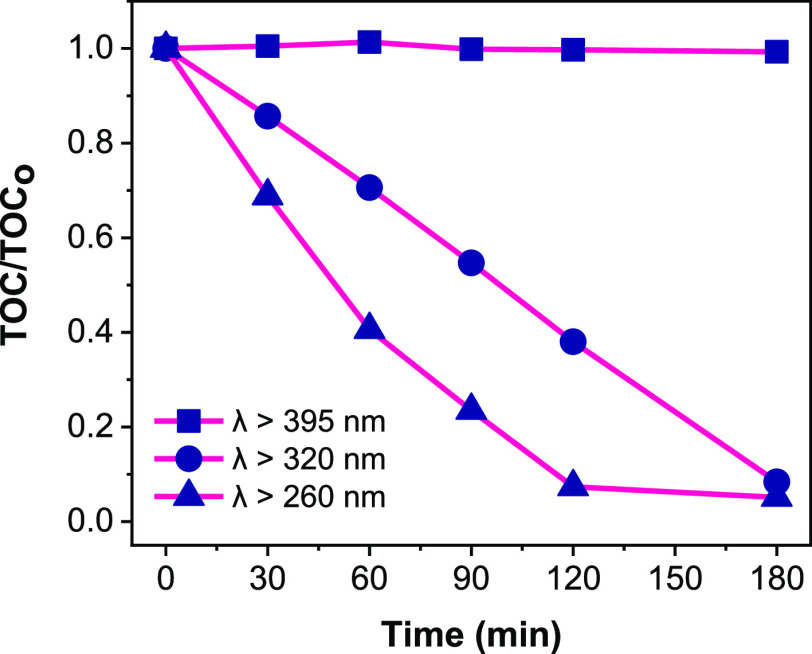
Mineralization profiles of 2,4,6-TCP using FeO_*x*_–CoO_*x*_/TiO_2_ sample under different light wavelength (λ) ranges. Conditions: 2,4,6-TCP, 50 ppm, 200 mL, pH = 6, catalyst concentration = 0.5 g/L.

The significant improvement in 2,4,6-TCP mineralization efficiency by FeO_*x*_–CoO_*x*_ compared to FeO_*x*_–CuO_*x*_ as bi-co-catalysts, as shown in Figure 2d, which is likely due to a high synergistic effect between FeO_*x*_ and CoO_*x*_ as good oxidation cocatalysts, thus resulting in improved electron/hole charge separation and generation of hydroxyl radicals for 2,4,6-TCP mineralization. The experimental error bar plot of the optimized FeO_*x*_–CoO_*x*_/TiO_2_ is shown in Figure S7 after carrying out the 2,4,6-TCP mineralization experiment in triplicates. The result signifies that the experiment is repeatable to a large extent. The photocatalytic activity of the optimized FeO_*x*_–CoO_*x*_/TiO_2_ sample was further evaluated under different light wavelengths and results are displayed in Figure 4. Virtually 0% TOC removal was observed after 3 h of light irradiation with a 395 nm cutoff filter. The outcome was not surprising, since surface impregnation of TiO_2_ with cocatalysts does not change the band gap energy of the PC50 TiO_2_ (ca. 3.2 eV) as represented in Figure 1a, which corresponds to light wavelength of around 390 nm. This further implies that the pristine and low concentration metal oxides-modified TiO_2_ samples cannot absorb light of wavelength above 390 nm, which results into negligible 2,4,6-TCP degradation activity. However, with light irradiation of wavelength (λ > 260 nm), there is a remarkable enhancement in the 2,4,6-TCP mineralization rate in the first 2 h, compared to that of light irradiation of wavelength (λ > 320 nm). This is due to the availability of stronger UV light (UV–B) irradiation and contributions from 2,4,6-TCP photodegradation, since 2,4,6-TCP absorbs UV light below 320 nm.^12^ Around 62% TOC removal was achieved with λ > 320 nm, while about 93% TOC removal was achieved with λ > 260 nm in 2 h.

**Figure 5:**
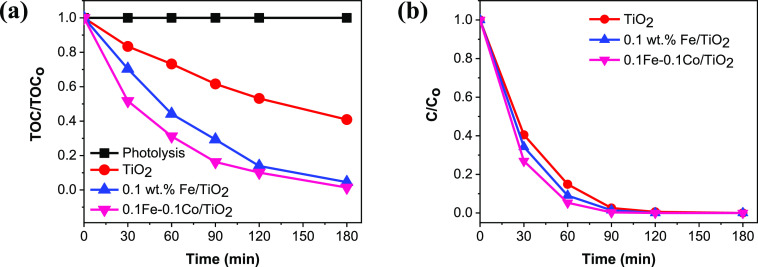
(a) Mineralization profiles of 2,4-D using PC50 TiO_2_, FeO_*x*_/TiO_2_ and FeO_*x*_–CoO_*x*_/TiO_2_ samples. (b) Conversion profiles of 2,4-D using PC50 TiO_2_, FeO_*x*_/TiO_2_, and FeO_*x*_–CoO_*x*_/TiO_2_ samples monitored by HPLC. Conditions: 2,4-D, 25 ppm, 200 mL, pH = 4, catalyst concentration = 0.5 g/L, λ > 320 nm.

Further studies on photocatalytic mineralization ability of the optimized FeO_*x*_–CoO_*x*_/TiO_2_ sample was evaluated with another widely used herbicide, 2,4-dichlorophenoxyacetic acid (2,4-D) and results are displayed in Figure 5a. Here, 0% TOC removal was observed after 3 h of light irradiation in the absence of a photocatalyst. Nearly 60%, 95%, and 100% TOC removal are achieved with the unmodified TiO_2_, FeO_*x*_/TiO_2_, and FeO_*x*_–CoO_*x*_/TiO_2_ samples in 3 h, respectively. 2,4-D removal by the optimized FeO_*x*_–CoO_*x*_/TiO_2_ was also analyzed with HPLC, and results are shown in Figure 5b. The 2,4-D degradation rates well agree with the corresponding mineralization rates in the first 60 min. The photocatalytic 2,4-D degradation efficiencies in 60 min follow the order: TiO_2_ (85%) < FeO_*x*_/TiO_2_ (91%) < FeO_*x*_–CoO_*x*_/TiO_2_ (95%). Beyond 60 min, the three samples recorded approximately similar photocatalytic activity for 2,4-D degradation. This is likely due to the presence of very low concentration of 2,4-D in solution and on surface of the photocatalyst. Also, the 2,4-D degradation process is very fast over FeO_*x*_ and FeO_*x*_–CoO_*x*_-decorated TiO_2_ samples compared to the slow mineralization process, since partial oxidation precedes the cleavage of the aromatic benzene ring to liberate carbon dioxide (CO_2_).^36−38^ The FeO_*x*_–CoO_*x*_/TiO_2_ sample also shows the highest 2,4-D mineralization efficiency but similar 2,4-D degradation performance compared to FeO_*x*_/TiO_2_ and TiO_2_. The experimental error bar plot of optimized FeO_*x*_–CoO_*x*_/TiO_2_ is also shown in Figure S8 after carrying out the 2,4-D mineralization experiment in triplicates. The result shows that the experiment is highly reproducible.

**Figure 6:**
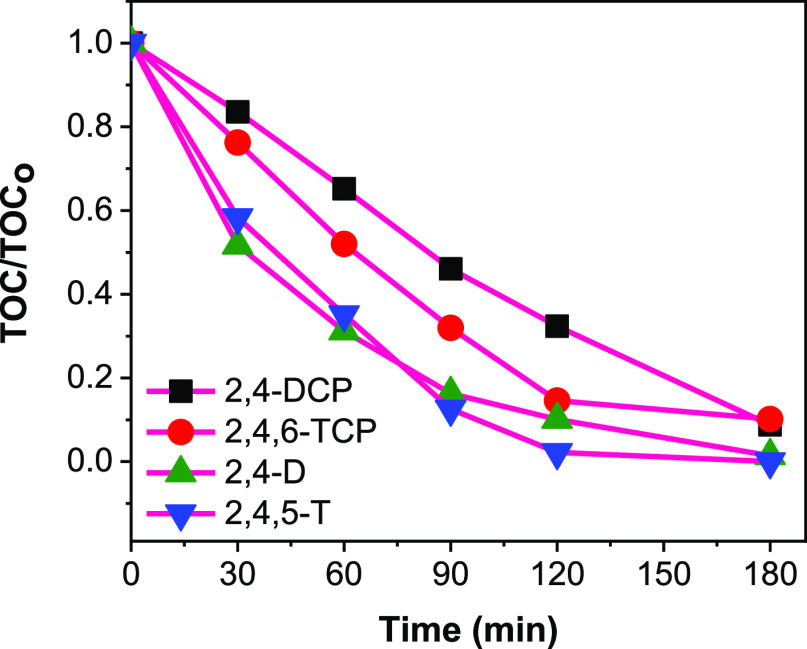
Mineralization profiles of selected herbicides using FeO_*x*_–CoO_*x*_/TiO_2_ sample under similar operating conditions. Conditions: Herbicides, 25 ppm, 200 mL, pH = 4, catalyst concentration = 0.5 g/L, λ > 320 nm; 2,4-dichlorophenol (2,4-DCP), 2,4,6-trichlorophenol (2,4,6-TCP), 2,4-dichlorophenoxyacetic acid (2,4-D), 2,4,5-trichlorophenoxyacetic acid (2,4,5-T).

In order to investigate how the chlorine substituents and other functional groups on the aromatic benzene ring influence photocatalytic degradation activity, two chlorophenols (2,4-DCP and 2,4,6-TCP) and two chlorinated herbicides (2,4-D and 2,4,5-T) were evaluated using the optimized photocatalyst composite. It was reported that the number and positions of the chlorine substituents play vital roles in predicting the level of toxicity and degradation rate of each members of the chlorinated phenols group.^46−50^ Based on the results in Figure 6, the two chlorophenols are more difficult to mineralize compared to their phenoxyacetic acid counterparts. This could be due to the difference in the oxygen functional groups (hydroxyl and acetic acid) on the aromatic ring for both classes of chlorinated herbicides, and likely the acetic acid group can be readily degraded. It is widely reported that 2,4,6-TCP degrades faster than 2,4-DCP during photocatalytic water treatment, consistent with our results in Figure 6.^48−50^ However, there is no clear relationship between the degradation rate of chlorophenols and number of chlorine substituents on the aromatic ring, but the position of Cl atoms was reported to highly determine the order of initial degradation rates.^51,52^ In the first 90 min, 2,4-DCP is the most difficult to mineralize, while 2,4-D and 2,4,5-T (with similar trend) are the easiest to mineralize. The temporal UV–vis absorption spectra of the four herbicides at 90 min of photocatalytic degradation on FeO_*x*_–CoO_*x*_/TiO_2_ are shown in Figure S9a–d. The results corroborate the earlier observation with TOC removal rates as 2,4-DCP remains the most difficult to degrade, while 2,4-D and 2,4,5-T are easiest to degrade.

### 3.3 Catalyst Recycling and Reactive Oxygen Species Tests

**Figure 7:**
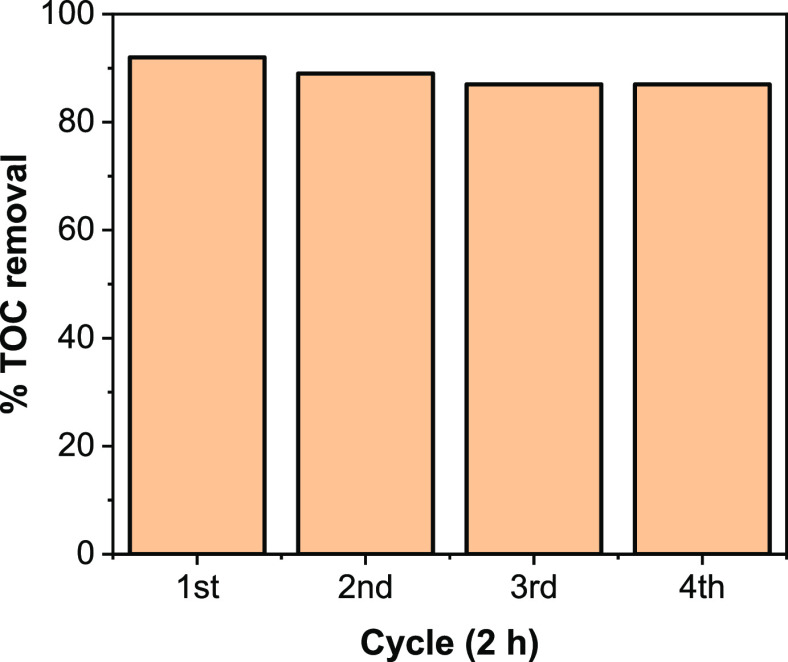
Recycling performance of FeO_*x*_–CoO_*x*_/TiO_2_ for 2,4,6-TCP mineralization. Conditions: 2,4,6-TCP, 50 ppm, 200 mL, pH = 6, catalyst concentration = 0.5 g/L, λ > 260 nm.

The stability of the best FeO_*x*_–CoO_*x*_/TiO_2_ composite photocatalyst was evaluated for 2,4,6-TCP mineralization under full arc light irradiation as shown in Figure 7. It can be seen that the photocatalytic activity of composite did not decrease conspicuously after four successive cycles (2 h) of 2,4,6-TCP mineralization test, indicating that the composite is fairly stable. The fair stability of FeO_*x*_–CoO_*x*_/TiO_2_ after four successive activity cycles suggests that a small amount of CoO_*x*_ is required in the FeO_*x*_–CoO_*x*_/TiO_2_ composite to improve its performance.

Furthermore, the loading metal content on fresh and used as-prepared samples was investigated by ICP-AES. As shown in Figure S10a–c, it can see that the actual Fe loading amount on three TiO_2_-based samples is quite close to the nominal value of 0.1 wt %. However, the actual Cu and Co loading amount are far less than the designed value, possibly due to the loss during the washing procedure. The metal leaching on various photocatalysts in photocatalytic 2,4,6-TCP mineralization after recycling experiments (2 h) was then studied. It is found that approximately 64.4 wt % of Fe on FeO_*x*_/TiO_2_ was leached (Figure S10a), probably because of the severe photocorrosion. Similar phenomenon happened on FeO_*x*_–CuO_*x*_/TiO_2_ (Figure S10b), where around 65.3 wt % of Fe and 71.2 wt % of Cu were leached respectively during the photocatalytic process. Nevertheless, the loss of Fe and Co was greatly reduced on FeO_*x*_–CoO_*x*_/TiO_2_ (S10c). Only 51 wt % of Fe and 3.6 wt % of Co are found to be leached, indicating the good stability of the optimized FeO_*x*_–CoO_*x*_/TiO_2_ in photocatalytic 2,4,6-TCP mineralization. Further study to improve the binding force of Fe on TiO_2_ is underway.

**Figure 8:**
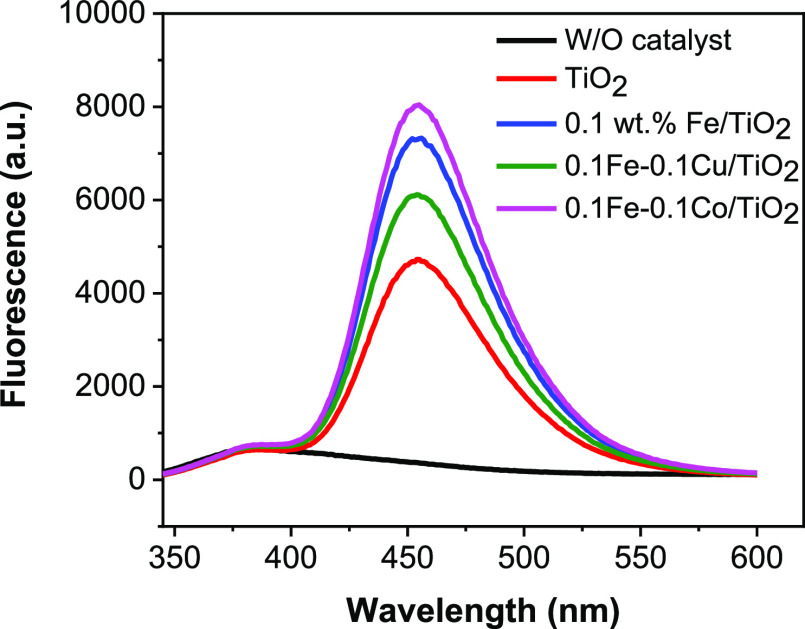
Fluorescence spectral changes observed during illumination of prepared TiO_2_ samples in 0.001 M aqueous solution of coumarin (excitation at 332 nm). Each fluorescence spectrum was recorded after 5 min of light illumination with multichannel 300 W UV 365 nm LED.

**Scheme 1:**
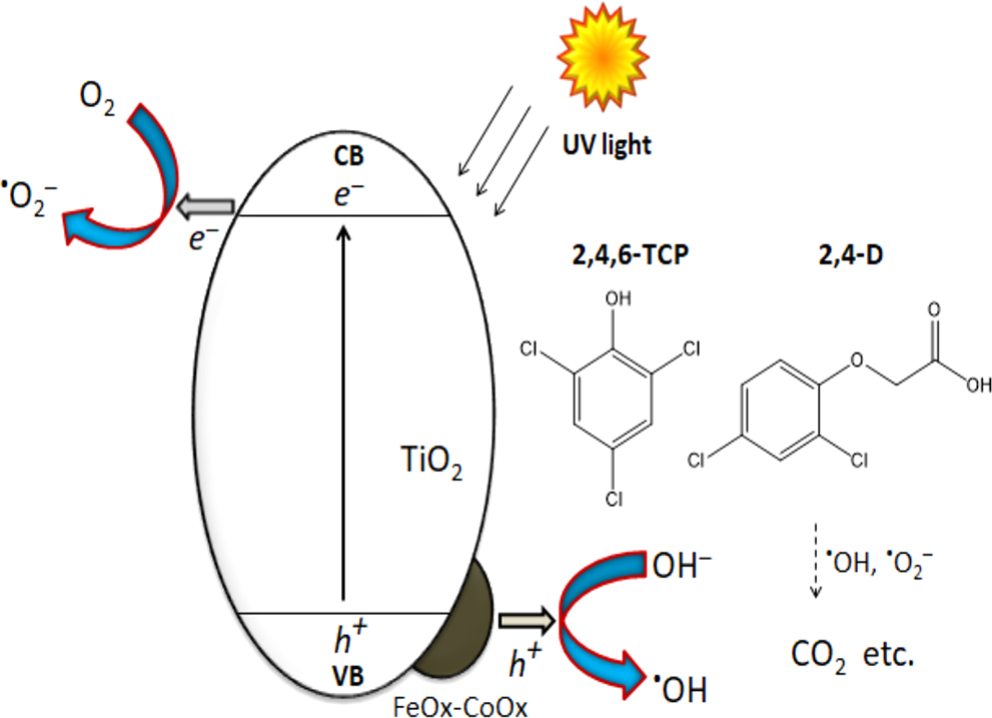
Proposed Mechanism for Major Charge Transfer Pathways on FeO_*x*_–CoO*_x_*/TiO_2_ for Mineralization of 2,4,6-TCP and 2,4-D

Hydroxyl radicals (^**•**^OH) are considered the major active species during photocatalytic degradation of organic water pollutants, while the short lifetime (∼10^–9^ s) and high reactivity of the hydroxyl radicals hinder its direct detection.^53,54^ Fluorescence spectroscopy was used to investigate the presence of ^**•**^OH with coumarin (COU) as a probe molecule (poor fluorescent dye). The COU can react with ^**•**^OH to give a highly fluorescent 7-hydroxycoumarin (7-HC).^54^ A very low concentration (10^–3^–10^–4^ M) of the probe molecule is often used during spectrofluorometric analysis to limit contributions from the valence band holes of TiO_2_ during illumination with UV light.^55^ Lower concentrations of the probe molecule usually favor hydroxylation reaction with ^**•**^OH.^55,56^Figure 8 shows the fluorescence spectra of COU solutions (0.001 M) containing 0.6 mg/mL of PC50 TiO_2_, FeO_*x*_/TiO_2_, FeO_*x*_–CuO_*x*_/TiO_2_, and FeO_*x*_–CoO_*x*_/TiO_2_ under 5 min irradiation from a multichannel 300 W UV LED (365 nm). No signal was observed without a photocatalyst. The fluorescent intensities at around 455 nm follow the order: PC50 TiO_2_ < FeO_*x*_–CuO_*x*_/TiO_2_ < FeO_*x*_/TiO_2_ < FeO_*x*_–CoO_*x*_/TiO_2_. The highest fluorescence intensity obtained from FeO_*x*_–CoO_*x*_/TiO_2_ implies a high concentration of 7-HC, as a result of high generation rate of ^**•**^OH radical, which is very beneficial for the degradation of these herbicides. This indicates that FeO_*x*_–CoO_*x*_/TiO_2_ facilitates charge separation via hole transfer from TiO_2_ as highlighted in the proposed semiconductor charge separation mechanism (Scheme 1). When the FeO_*x*_–CoO_*x*_/TiO_2_ photocatalyst is exposed to ultraviolet light, photogenerated electrons are excited from the valence band (VB) to the conduction band (CB) of TiO_2_. The VB holes are transferred to the surface FeO_*x*_/CoO_*x*_ sites and subsequently react with hydroxyl ions (OH^–^) in water to produce OH radicals, which oxidize 2,4,6-TCP and other herbicides to intermediate products before their mineralization. At the same time, photoelectrons are consumed by oxygen to produce superoxides that react with the intermediates to form the final CO_2_ and water.

## 4 Conclusion

In summary, a facile and robust synthesis procedure was successfully used in decorating PC50 TiO_2_ nanoparticles with highly dispersed mono and dual cocatalysts (FeO_*x*_, CuO_*x*_, CoO_*x*_, FeO_*x*_–CuO_*x*_, and FeO_*x*_–CoO_*x*_). The binary cocatalyst comprising FeO_*x*_–CoO_*x*_ plays a key role for efficient decomposition of widely used four chlorinated herbicides. FeOOH (denoted as FeO_*x*_ in this article), CuO and CoO nanoparticles were determined to be the only decorating species. The highest photocatalytic 2,4,6-TCP mineralization efficiency has been achieved by the FeO_*x*_–CoO_*x*_/TiO_2_. About 92% 2,4,6-TCP mineralization and 100% 2,4,6-TCP degradation efficiencies in 3 h are achieved over the optimized FeO_*x*_–CoO_*x*_/TiO_2_ sample, which is about three times higher than the benchmark reference PC50 TiO_2_. The optimized FeO_*x*_–CoO_*x*_/TiO_2_ sample also exhibits 150% activity enhancement for another herbicide (2,4-D) degradation compared to TiO_2_. However, the FeO_*x*_–CoO_*x*_/TiO_2_ sample shows negligible activity for 2,4,6-TCP degradation under visible light (λ > 395 nm) irradiation. This is primarily due to the UV absorption bandgap energy of PC50 TiO_2_ (3.2 eV or 390 nm), since the low concentration surface cocatalysts do not alter its bandgap based on the UV absorption spectra of the prepared photocatalysts. Under similar operating conditions, the mineralization rates with 2,4-D and 2,4,5-T are higher compared to 2,4-DCP and 2,4,6-TCP. 2,4-DCP is the most difficult to mineralize. The observation is likely due to the nature of oxygen functional groups and the relative position of Cl substituents on the aromatic ring. The enhancement in photocatalytic degradation four herbicides over the optimized FeO_*x*_–CoO_*x*_/TiO_2_ sample is likely due to the improved change separation, hole accumulation on FeO_*x*_–CoO_*x*_, and enhanced hydroxyl radicals generation as FeO_*x*_–CoO_*x*_ clusters are an excellent hole acceptor. Finally the optimized photocatalyst was proved to be rather stable for these herbicides’ mineralization.

## Supporting Information Available

Figure S1, full XPS survey spectra of FeO_*x*_–CuO_*x*_/TiO_2_ and FeO_*x*_–CoO_*x*_/TiO_2_; Figure S2, Co 2p XPS spectra of FeO_*x*_–CoO_*x*_/TiO_2_; Figure S3, Fe 2p XPS spectra of FeO_*x*_–CoO_*x*_/TiO_2_; Figure S4, Ti 2p XPS spectra of TiO_2_ based samples; Figure S5, mineralization profile of 2,4,6-TCP on FeO_*x*_ or/and CuO_*x*_ loaded TiO_2_; Figure S6, ineralization profile of 2,4,6-TCP on FeO_*x*_ or/and CoO_*x*_ loaded TiO_2_; Figure S7, TOC files with error bar on FeO_*x*_–CoO_*x*_/TiO_2_ for 2,4,6-TCP mineralization; Figure S8, TOC files with error bar on FeO_*x*_–CoO_*x*_/TiO_2_ for 2,4-DCP mineralization; Figure S9, temporal UV–vis adsorption spectra of 2.4-DCP, 2,4,6-TCP, 2,4-D, and 2,4,5-T on FeO_*x*_–CoO_*x*_/TiO_2_ in photocatalytic degradation; and Figure S10, metal loading amount on fresh and used TiO_2_-based samples detected by ICP-AES. (PDF)
