# Supplementary material for: Bimetallic FeOx–MOx Loaded TiO2 (M = Cu, Co) Nanocomposite Photocatalysts for Complete Mineralization of Herbicides
Source: J Phys Chem C Nanomater Interfaces. 2023 Jan 17;127(3):1388–96. doi: 10.1021/acs.jpcc.2c06796 (PMC9884081; doi:10.1021/acs.jpcc.2c06796)
Supplement: Supplementary file 1 — jp2c06796_si_001.pdf [file jp2c06796_si_001.pdf]

Supporting Information (SI) for Publication

Bimetallic FeO_x-MO_x Loaded TiO_2 (M = Cu, Co) Nanocomposite Photocatalysts for Complete Mineralisation of Herbicides

Ayoola Shoneye[a], Haimiao Jiao[a] and Junwang Tang*[a]

[a] A. Shoneye, H. Jiao and Prof. J. Tang
Department of Chemical Engineering
University College London
Torrington Place, London, WC1E 7JE, UK.
E-mail: junwang.tang@ucl.ac.uk

Table of contents

Figure S1. Full XPS survey spectra of FeO_x-CuO_x/TiO_2 and FeO_x-CoO_x/TiO_2 samples with optimum co-catalyst loading (0.1 wt.% for each metal).

Figure S2. Co 2p XPS spectra of FeO_x-CoO_x/TiO_2 sample with optimum Co loading (0.1 wt.% Co).

Figure S3. Fe 2p XPS spectra of FeO_x-CoO_x/TiO_2 sample with optimum Fe loading (0.1 wt.% Fe).

Figure S4. Ti 2p XPS spectra of co-catalyst(s)-loaded TiO_2.

Figure S5. Mineralisation profiles of 2,4,6-TCP on FeO_x/TiO_2, CuO_x/TiO_2 and FeO_x-CuO_x/TiO_2.

Figure S6. Mineralisation profiles of 2,4,6-TCP on FeO_x/TiO_2, CoO_x/TiO_2 and FeO_x-CoO_x/TiO_2.

Figure S7. TOC measurements with experimental error bar on FeO_x-CoO_x/TiO_2 for 2,4,6-TCP mineralisation after conducting triple experiments.

Figure S8. TOC measurements with experimental error bar on FeO_x-CoO_x/TiO_2 for 2,4-D mineralisation after conducting triple experiments.

Figure S9. **(a)** Temporal UV-vis absorption spectra of 2,4-dichlorophenol (2,4-DCP) at 90 minutes of photocatalytic degradation, **(b)** Temporal UV-vis absorption spectra of 2,4,6-trichlorophenol (2,4,6-TCP) at 90 minutes of photocatalytic degradation, **(c)** Temporal UV-vis absorption spectra of 2,4-dichlorophenoxyacetic acid (2,4-D) at 90 minutes of photocatalytic degradation, **(d)** Temporal UV-vis absorption spectra of 2,4,5-trichlorophenoxyacetic acid (2,4,5-T) at 90 minutes of photocatalytic degradation. FeO_x-CoO_x/TiO_2 was used in this study.

Figure S10. The different metal loading amount detected by ICP-AES on fresh and used **(a)** FeO_x/TiO_2, **(b)** FeO_x-CuO_x/TiO_2 and **(c)** FeO_x-CoO_x/TiO_2.

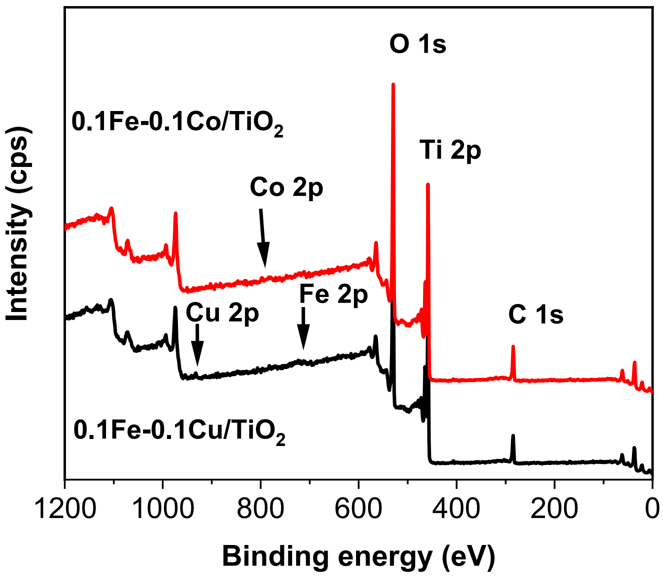

Figure S1. Full XPS survey spectra of FeO_x-CuO_x/TiO_2 and FeO_x-CoO_x/TiO_2 samples with optimum co-catalyst loading (0.1 wt.% for each metal).

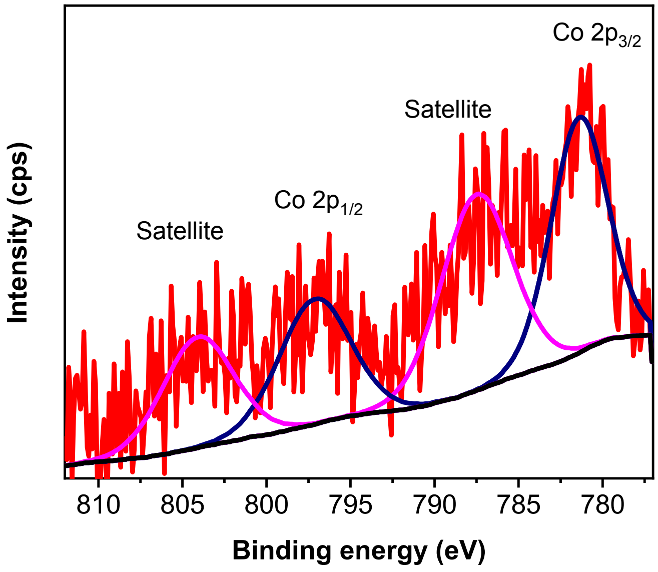

Figure S2. Co 2p XPS spectra of FeO_x-CoO_x/TiO_2 sample with optimum Co loading (0.1 wt.% Co).

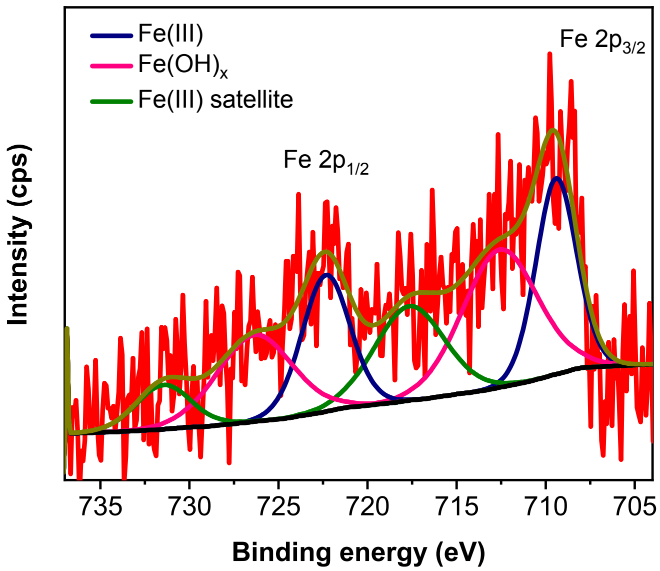

Figure S3. Fe 2p XPS spectra of FeO_x-CoO_x/TiO_2 sample with optimum Fe loading (0.1 wt.% Fe).

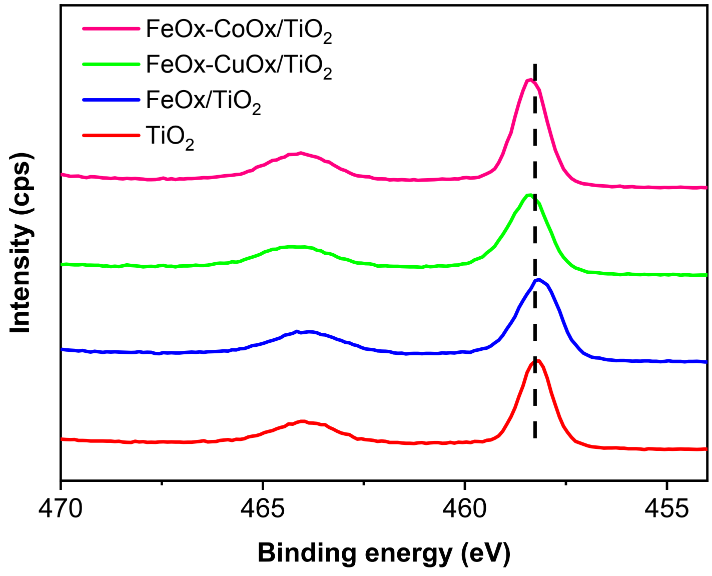

Figure S4. Ti 2p XPS spectra of co-catalyst(s)-loaded TiO_2.

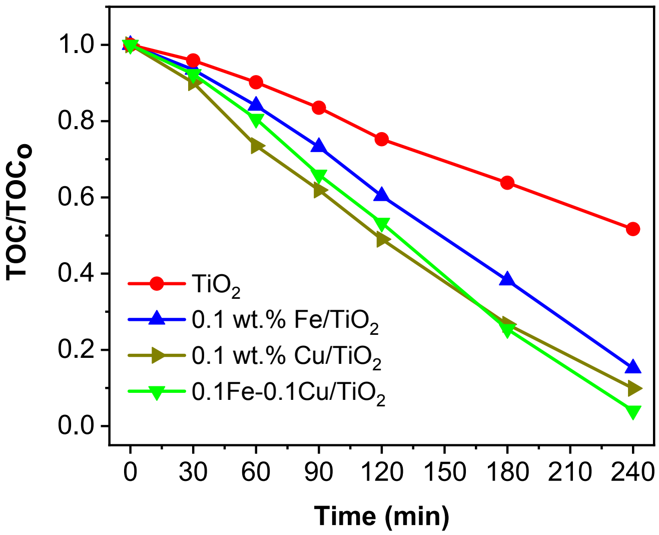

Figure S5. Mineralisation profiles of 2,4,6-TCP on FeO_x/TiO_2, CuO_x/TiO_2 and FeO_x-CuO_x/TiO_2.

Conditions: 2,4,6-TCP (50 ppm, 200 mL, pH = 6, catalyst concentration = 0.5 g/L, λ > 320 nm).

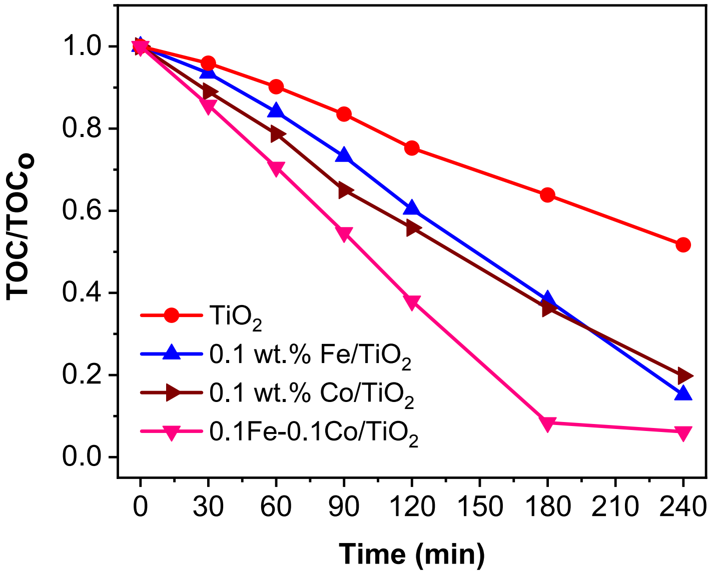

Figure S6. Mineralisation profiles of 2,4,6-TCP on FeO_x/TiO_2, CoO_x/TiO_2 and FeO_x-CoO_x/TiO_2.

Conditions: 2,4,6-TCP (50 ppm, 200 mL, pH = 6, catalyst concentration = 0.5 g/L, λ > 320 nm).

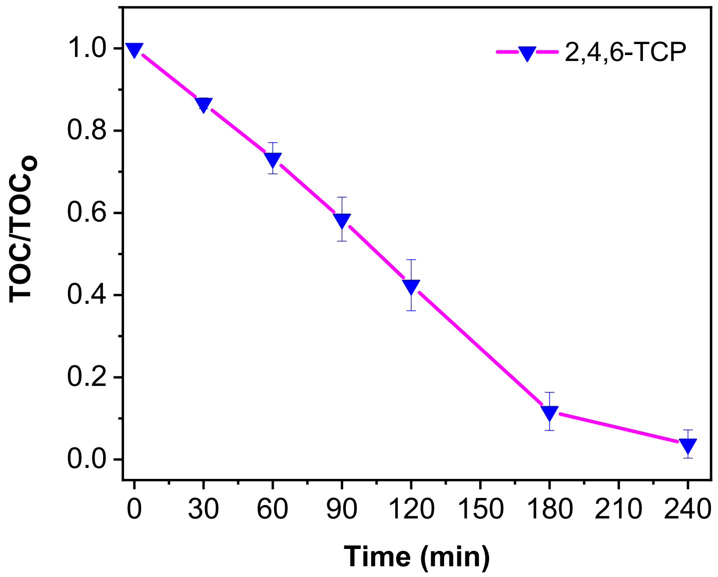

Figure S7. TOC measurements with experimental error bar on FeO_x-CoO_x/TiO_2 for 2,4,6-TCP mineralisation after conducting triple experiments.

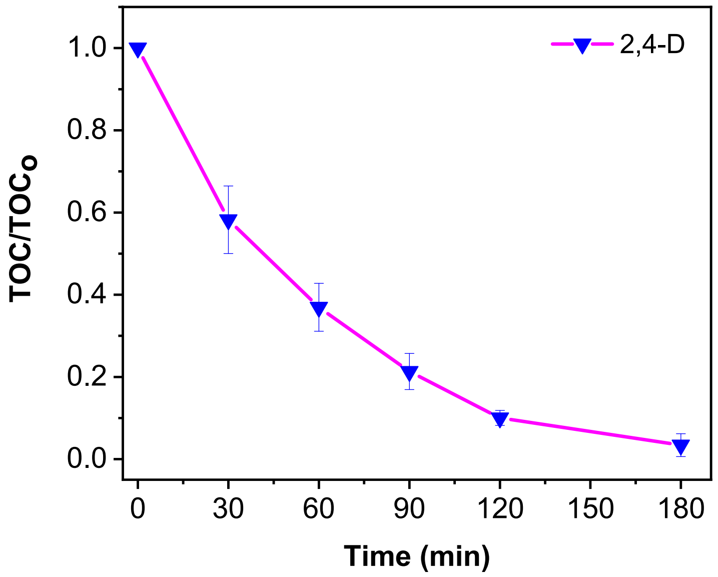

Figure S8. TOC measurements with experimental error bar on FeO_x-CoO_x/TiO_2 for 2,4-D mineralisation after conducting triple experiments.

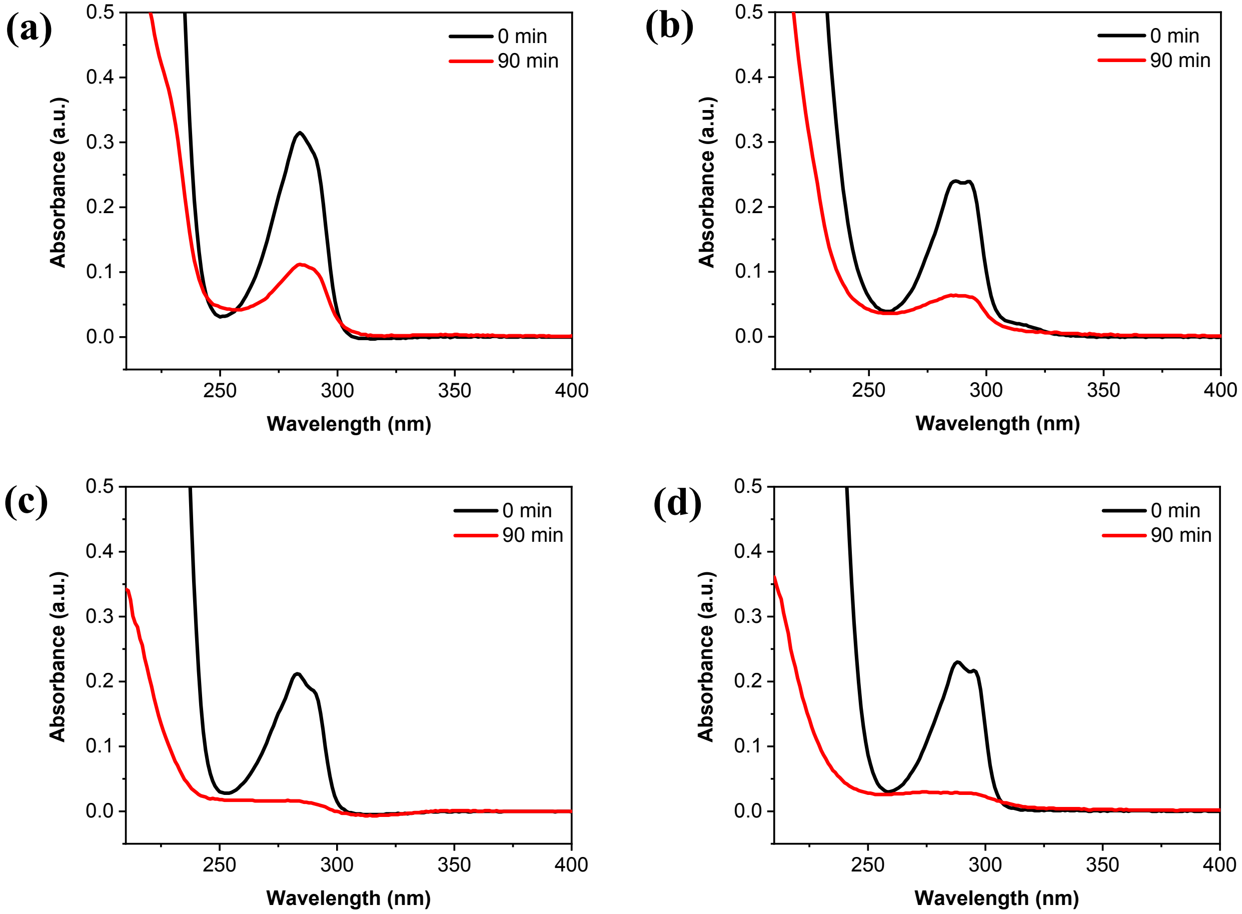

Figure S9. (a) Temporal UV-vis absorption spectra of 2,4-dichlorophenol (2,4-DCP) at 90 minutes of photocatalytic degradation, **(b)** Temporal UV-vis absorption spectra of 2,4,6-trichlorophenol (2,4,6-TCP) at 90 minutes of photocatalytic degradation, **(c)** Temporal UV-vis absorption spectra of 2,4-dichlorophenoxyacetic acid (2,4-D) at 90 minutes of photocatalytic degradation, **(d)** Temporal UV-vis absorption spectra of 2,4,5-trichlorophenoxyacetic acid (2,4,5-T) at 90 minutes of photocatalytic degradation. FeO_x-CoO_x/TiO_2 was used in this study.

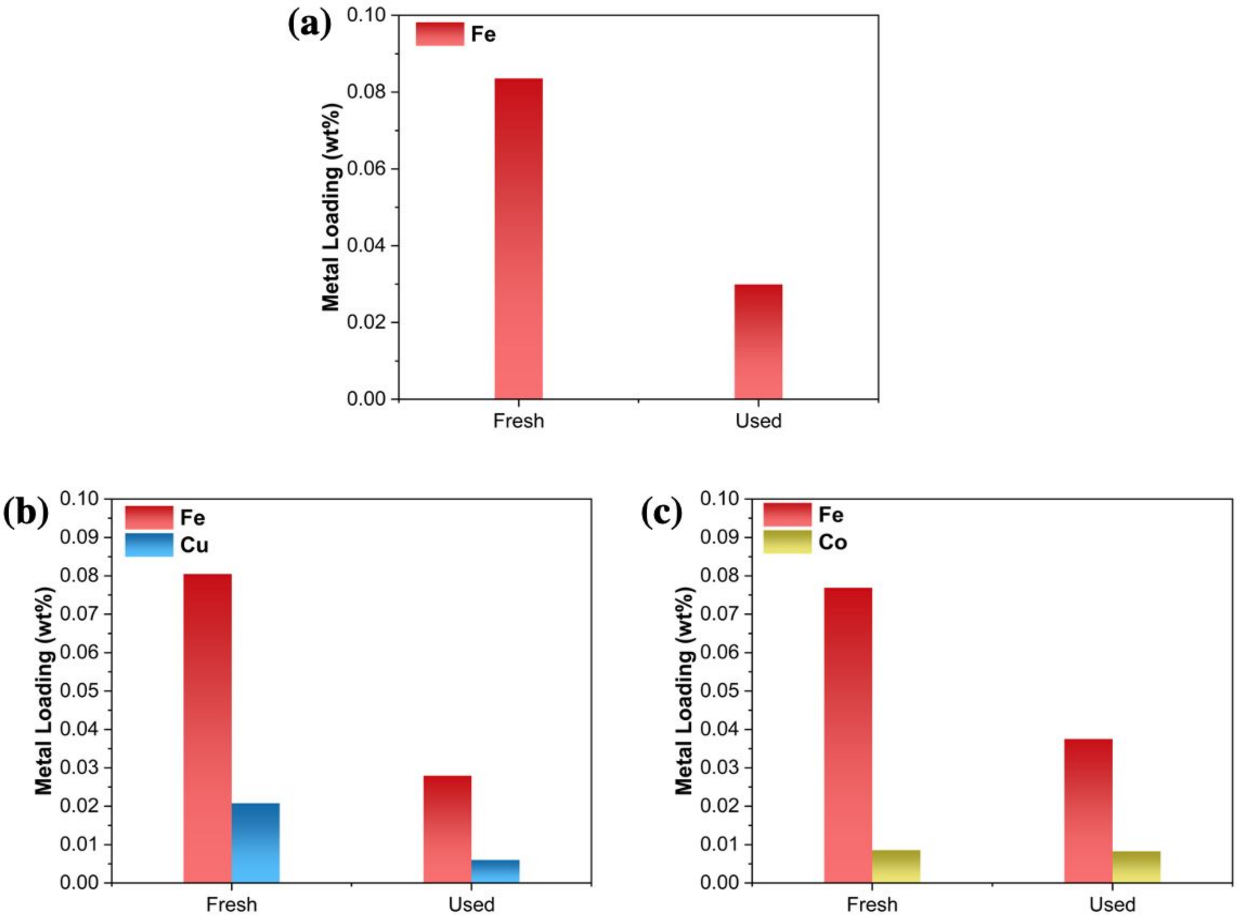

Figure S10. The different metal loading amount detected by ICP-AES on fresh and used **(a)** FeO_x/TiO_2, **(b)** FeO_x-CuO_x/TiO_2 and **(c)** FeO_x-CoO_x/TiO_2.
